# Maternal Pea Protein Intake Provides Sex-Specific Protection against Dyslipidemia in Offspring from Obese Pregnancies

**DOI:** 10.3390/nu15040867

**Published:** 2023-02-08

**Authors:** Todd C. Rideout, Gabriella A. Andreani, Jillian Pembroke, Divya Choudhary, Richard W. Browne, Saleh Mahmood, Mulchand S. Patel

**Affiliations:** 1 Department of Exercise and Nutrition Sciences, School of Public Health and Health Professions, University at Buffalo, Buffalo, NY 14214, USA; 2 Department of Biotechnical and Clinical Laboratory Sciences, Jacobs School of Medicine and Biomedical Sciences, University at Buffalo, Buffalo, NY 14214, USA; 3 Department of Biochemistry, Jacobs School of Medicine and Biomedical Sciences, University at Buffalo, Buffalo, NY 14214, USA

**Keywords:** maternal obesity, offspring, yellow pea protein, lipids, rats, dietary pulses

## Abstract

Increased consumption of dietary pulse protein has been shown to assist in body weight regulation and improve a range of metabolic health outcomes. We investigated if the exchange of casein for yellow pea protein (YPPN) in an obese-inducing maternal diet throughout pregnancy and lactation offered protection against obesity and dyslipidemia in offspring. Sixty female Sprague Dawley rats were fed a low-calorie control diet (CON), a high-caloric obesity-inducing diet (with casein protein (CP), HC-CP), or an isocaloric/macronutrient-matched HC diet supplemented with YPPN isolate (HC-PPN) in pre-pregnancy, gestation, and lactation. Body weight (BW) and metabolic outcomes were assessed in male and female offspring at weaning and in adulthood after consuming the CON diet in the postnatal period. Consumption of the HC-PPN diet did not protect against maternal obesity but did improve reproductive success compared with the HC-CP group (72.7% versus 43.7%) and reduced total energy, fat, and protein in maternal milk. Male, but not female, offspring from mothers fed the HC-CP diet demonstrated hyperphagia, obesity, dyslipidemia, and hepatic triglyceride (TG) accumulation as adults compared with CON offspring. Isocaloric exchange of CP for YPPN in a high-calorie obese-inducing diet did not protect against obesity but did improve several aspects of lipid metabolism in adult male offspring including serum total cholesterol, LDL/VLDL cholesterol, triglycerides (TGs), and hepatic TG concentration. Our results suggest that the exchange of CP for YPPN in a maternal obese-inducing diet selectively protects male offspring from the malprogramming of lipid metabolism in adulthood.

## 1. Introduction

With an alarming prevalence of ~40% among American adults, obesity is a critical healthcare priority as it is closely linked with metabolic dysfunction (e.g., insulin resistance and dyslipidemia) and considered a ‘gateway’ disease that substantially increases the risk of diabetes, cardiovascular disease (CVD), and cancer [1,2]. The etiology of obesity is complex, but with the recent increase in childhood obesity, there has been a re-evaluation of early-life in utero and immediate postnatal factors that may influence lifelong obesity risk. Approximately fifty percent of pregnant women in the United States are overweight or obese (3), putting not only the mothers’ health at risk but also placing a substantial health burden on future generations before they are even born. Maternal obesity fosters an adverse in utero environment and can further influence immediate postnatal nutrient and hormone exposure through altered maternal milk composition [3,4], strongly shaping fetal development and early childhood health [5]. Offspring from obese mothers exhibit a range of metabolic abnormalities including impaired regulation of appetite and energy expenditure [6], increased adiposity [7], reduced glycemic control [8], and dyslipidemia [9].

Maternal nutrition before, during, and after pregnancy is instrumental in ensuring early-life health and shaping lifelong disease risk trajectories in offspring [10]. However, nutrition amongst women of child-bearing age is suboptimal, with nutrient and calorie intake often exceeding recommendations for energy, sugar, and saturated fat and being below recommendations for micronutrients [11,12,13]. Consumption of ultra-processed foods during pregnancy has been associated with lower diet quality, including in terms of total and plant-based proteins [14]. Pregnant mothers require a higher protein intake to support fetal growth and development, and both the source and amount of dietary protein may affect pregnancy outcomes and have implications for the long-term health of offspring [15,16].

Dietary pulses, including dry beans, peas, and lentils, have an outstanding nutritional profile and are a rich source of protein (~7.7 g of protein in ½ a cup) [17]. Supplementation with pulse protein isolates, which contain not only high-quality protein but also an array of functional bioactive compounds (i.e., phenols and bioactive peptides), has been shown to have health benefits in previous rodent studies, including protection against body weight (BW) gain, increased cecal short-chain fatty acid production, and reduced blood pressure and serum cholesterol [18,19,20,21]. However, we are not aware of previous work that has examined maternal consumption of pulse proteins in obese pregnancies as a potential strategy to improve both maternal and offspring health. Therefore, the objective of this study was to examine if the exchange of casein for yellow pea protein (YPPN) in an obese-inducing maternal diet throughout pregnancy and lactation could influence pregnancy outcomes and offer protection against obesity and dyslipidemia in offspring.

## 2. Materials and Methods

**Figure 1:**
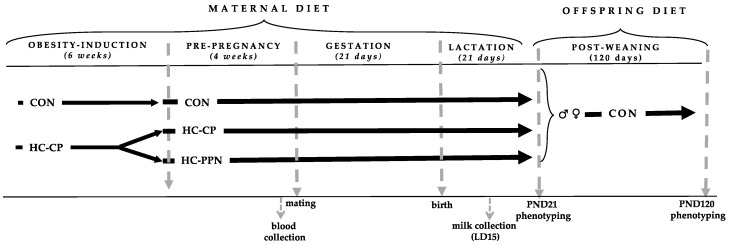
Experimental design. CON, low-calorie control diet; HC-CP, high-calorie obese-inducing diet with casein protein; HC-PPN, HC high-calorie diet with supplemental yellow pea protein (25%); LD, lactation day; and PND, postnatal day.

**Animals, diets, and design:** The experimental design is presented in Figure 1. Sixty newly-weaned [postnatal day (PND 21] female Sprague Dawley rats (Charles River, obese prone, Crl:OP-CD) were brought to the Laboratory Animal Facility at the University at Buffalo and kept under controlled conditions of light (12 h light:12 h dark), temperature (18–23 °C), and humidity (50%), with free access to food and water.

Following a 1-week chow-fed acclimation period, the rats were randomized to 1 of 2 semi-purified diets for a 6-week obesity induction phase (Table 1) consisting of (i) a low-calorie control diet (CON; n = 10; total energy 3.8 kcal/g; % energy from fat, 10; protein, 20; and carbohydrate, 70) (Research Diets, D12450K) or (ii) a high-caloric obesity-inducing diet with casein protein (HC-CP; n = 50; total energy 4.8 kcal/g; % energy from fat, 44; protein, 20; and carbohydrate, 35) (Research Diets, D12451).

Following the 6-week obese-inducing phase, obese HC-CP animals demonstrating an increased BW of ≥20% vs. CON females were randomized to either remain on the HC-CP diet (n = 20) or be provided with the HC diet supplemented with the YPPN isolate at the expense of casein (HC-PPN, n = 15) (25%, Vitessence Pulse 1803 pea protein, Ingredion) for an additional 4 weeks prior to mating. The HC-PPN diet was formulated to be similarly matched for energy, macronutrient, and total fiber content to the HC-CP diet based on proximate nutrient analyses of the YPPN isolate (moisture, 9.4%; ash, 4.21%; protein, 71.48%; fat, 6.78%; total carbohydrate, 8.13%; and calories, 3.38 kcal/g).

At the end of the obese-inducing and pre-pregnancy periods (a total of 10 weeks), non-fasting tail vein blood was collected, and the rats were bred with CON-fed male breeders to establish a timed pregnancy [22]. Pregnancy was confirmed by the presence of vaginal plugs and/or spermatozoa in vaginal lavage. Maternal BW and food intake were collected weekly throughout gestation and lactation. Following delivery, litter size and weights were recorded, and the litters were adjusted to 8 pups per dam within 24 h after birth. Where possible, litters were equally matched for the number of males and females. Litter weights were recorded weekly throughout lactation.

On lactation day 15, maternal milk was collected at a fixed time, between the hours of 9:00 to 11:00 am [23,24]. Dams received an intraperitoneal injection of oxytocin (Aspen Veterinary Resources Ltd., 2 IU/kg BW) to stimulate milk secretion and separated from their pups for ~30 min. While under isoflurane anesthesia (3.5%), milk was collected in a 200 µL capillary tube following manual expression of the teat using a gentle massage. The milking procedure took ~15 min, at which point the mothers were returned to their litters.

At weaning on postnatal day 21 (PND 21), 6 offspring from each group (3 males and 3 females) were randomly selected for metabolic phenotyping in a non-fasted state. Following anesthetization, blood was collected via cardiac puncture and pooled by sex for serum separation and subsequent lipid analyses. Livers were quickly excised, weighed, flash frozen in liquid nitrogen, and stored at −80 °C for further processing and analyses.

The remaining pups (one male and one female) from each litter were weaned onto the CON diet until PND120. Food intake (ad libitum) and BW were monitored weekly throughout the post-weaning period. On PND120, adult offspring were anesthetized for non-fasting metabolic characterization as described above. The rats used in this experiment were cared for in accordance with the guidelines established by the Institutional Animal Care and Use Committee. All procedures were reviewed and approved by the Animal Care Committee at the University at Buffalo.

**Blood and milk biochemistry:** Maternal glucose was measured using colorimetric detection (Invitrogen, Frederick, MD, USA; EIAGLUC) and insulin using ELISA (Millipore, Billerica, MA, USA; EZRMI-13K). Serum cholesterol profiles (TC, LDL/VLDL-C, and HDL-C) in newly weaned and adult offspring were determined using enzymatic analysis (BioAssay Systems, Hayward, CA, USA; EHDL-100). Serum TG (adult offspring only) concentration was measured using enzymatic analysis (Zenbio, Durham, NC, USA; STG-1NC). Maternal milk was assessed for protein using the Bradford assay (Biorad, Hercules, CA, USA), fat using creamatocrit assessment, and carbohydrate using colorimetric analyses (Biovision, Waltham, MA, USA). Total energy content of the milk was estimated based on the analyzed concentrations of protein (4 kcal/mL), carbohydrate (4 kcal/mL), and fat (9 kcal/mL).

**Tissue lipid analyses:** For the assessment of offspring hepatic TG, 50–100 mg of frozen tissue was homogenized in an aqueous NP-40 (5%) solution, followed by heating at 90 °C for 10 **min** and centrifugation at 12,000× *g* for 2 min. TG concentration in the supernatant was measured with a commercial kit (Zenbio, STG-1-NC) according to the manufacturer’s instructions. Hepatic cholesterol was extracted and analyzed according to our previously published procedures [25,26]. Approximately 0.5 g of pulverized liver was spiked with α-cholestane as an internal standard and saponified in freshly prepared KOH–methanol at 100 °C for 1 h. The non-saponifiable sterol fraction was extracted with petroleum diethyl ether and dried under N_2_ gas. Sterol fractions were analyzed with a Shimadzu GC-17A gas chromatograph fitted with a flame ionization detector using a SAC-5 capillary column (30 m × 0·25 mm × 0·25 mm, Supelco, Bellefonte, CA, USA).

**mRNA extraction and real-time RT-PCR:** Total RNA was isolated from frozen pulverized liver tissue (~25 mg) using the RNeasy Mini Kit (Qiagen). RNA concentration and integrity were determined with spectrophotometry (260 nm) and agarose gel electrophoresis, respectively. RNA preparation and real-time RT-PCR were conducted using a one-step QuantiFast SYBR Green RT-PCR kit (Qiagen) with a Biorad CFX96 Touch real-time PCR system. Gene expression was analyzed using the 2(-delta delta *C*t) method. The following validated primer sets for target and housekeeping genes were purchased from Qiagen (QuantiTect Primer Assay): β-actin (*Actb,* GeneGlobe ID: QT00193473), fatty acid synthase (*Fasn*) (QT00371210), acetyl-CoA carboxylase (*Acaca*, QT00190946), sterol regulatory element-binding protein 1c (*Srebf1, QT00432684*), and carnitine palmitoyltransferase 1a (*Cpt1a, QT01798825*).

**Data analyses**: All statistical analyses were conducted using SPSS 16 (SPSS Inc, Chicago, IL). Data were checked for normality using the Shapiro–Wilk test. Maternal outcomes were measured with a one-way ANOVA with a least significant difference (LSD) post hoc test. Litters from each dam were considered as a single observation. The main effects of maternal exposure (CON, HC-CP, and HC-PPN) and sex (male and female from the same maternal exposure) and interaction-related effects were analyzed via two-way ANOVA. If a significant main effect or interaction was detected, a one-way ANOVA with an LSD post hoc test was conducted to assess the programming responses. Data are presented as means ± SE. Differences were considered significant at *p* < 0.05.

## 3. Results

**Maternal and pregnancy outcomes:** Maternal phenotype and pregnancy outcomes are presented in Table 2. Compared with CON dams, those consuming the HC-CP and HC-PPN diets demonstrated increased (*p* < 0.05) BWs and caloric intakes throughout pre-pregnancy and gestation, with no differences (*p* > 0.05) noted between the HC-CP and HC-PPN groups.

Although no difference (*p* > 0.05) was observed in time to pregnancy between groups, reproductive success, defined as mothers who gave birth to a live litter without subsequent infanticide, was reduced in HC-CP mothers (43.7%) versus CON mothers (90.0%) but improved (72.7%) in mothers consuming the HC-PPN diet. Litter size and weight and average pup weight at birth did not differ (*p* > 0.05) between treatment groups. Consumption of the treatment diets did not alter (*p* > 0.05) maternal glucose, insulin, or the glucose:insulin ratio by the end of pre-pregnancy.

**Figure 2:**
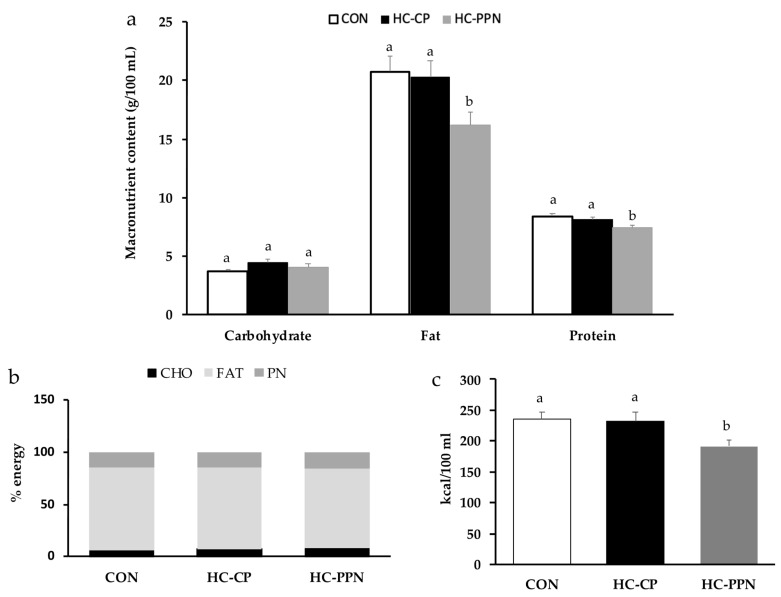
Nutrient content of maternal milk on lactation day 15. (**a**) Macronutrient content presented as g/100 mL; (**b**) macronutrient content presented as % energy; and (**c**) total energy content. CON, low-calorie control diet; HC-CP, high-calorie obese-inducing diet with casein protein; and HC-PPN, HC high-calorie diet with supplemental yellow pea protein (25%). ^ab^ Treatment groups within sex that do not share a superscript are significantly different (*p* < 0.05). Data are means ± SE, and n = 7–9 mothers per group.

**Maternal milk composition:** Compared with the CON and HC-CP groups, maternal milk from HC-PPN mothers had a lower (*p* < 0.05) fat and protein content (g/100 mL, Figure 2a) but no change (*p* > 0.05) in macronutrients when expressed as % energy (Figure 2b). Milk from HC-PPN mothers had a lower (*p* < 0.05) total energy content compared with milk from CON and HC-CP mothers (Figure 2c).

**Figure 3:**
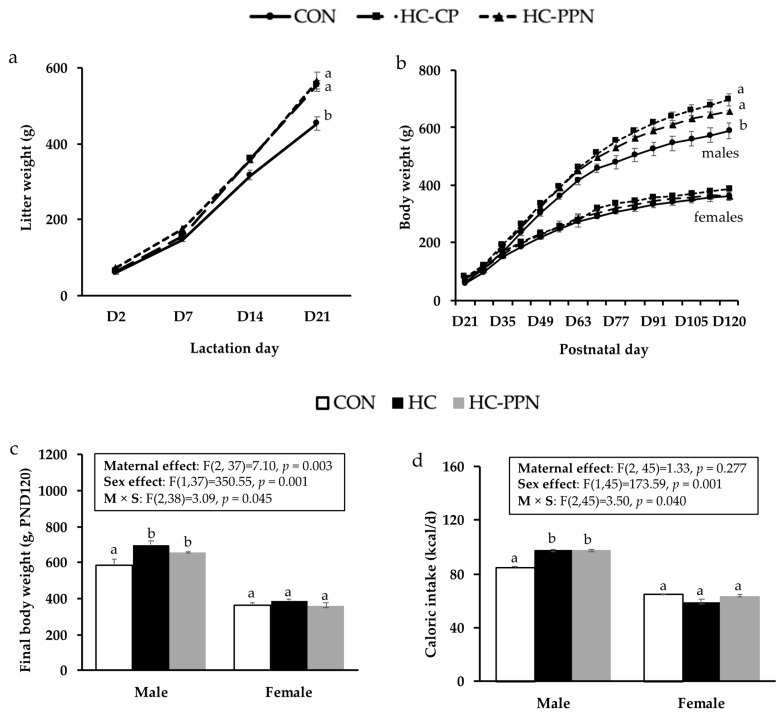
Offspring growth and feed intake. (**a**) Litter growth (g) throughout the lactation period; (**b**) postnatal male and female offspring growth trajectory (g); (**c**) final body weights (g) in male and female offspring; and (**d**) average postnatal caloric intake (kcal/d) in male and female offspring. CON, low-calorie control diet; HC-CP, high-calorie obese-inducing diet with casein protein; and HC-PPN, HC high-calorie diet with supplemental yellow pea protein (25%). Maternal (M), sex (S), and interaction (M × S) were analyzed using two-way ANOVA with LSD post hoc test (*p* < 0.05). ^ab^ Treatment groups within sex that do not share a superscript are significantly different (*p* < 0.05). Data are means ± SE, and n = 7–9 mothers per group.

**Post-weaning offspring growth and caloric intake**: Following the culling of litters, the trajectories of litter weights were increased (*p* < 0.05) in HC-CP and HC-PPN litters versus CON litters (Figure 3a). A significant maternal diet x sex effect was observed for final BW and feed intake in adult offspring. Adult male offspring from HC-CP and HC-PPN mothers had increased final BWs (Figure 3b,c) and caloric intakes (Figure 3d) versus offspring from CON mothers; however, no difference (*p* < 0.05) between these 2 groups was observed. Maternal diet did not influence BW or caloric intake in female offspring (Figure 3b–d).

**Offspring metabolic outcomes:** Newly weaned male and female offspring from HC-PPN dams demonstrated lower serum total-C compared with the CON offspring, mainly due to a reduction in HDL-C (Table 3). Total- and LDL/VLDL-C were increased (*p* < 0.05) in adult male offspring from HC-CP versus CON dams but reduced (*p* < 0.05) in HC-PPN offspring (vs. HC-CP). Serum TG was reduced (*p* < 0.05) in adult male HC-PPN offspring compared with CON and HC-CP offspring; however, no effect was observed in adult females (Table 3).

**Figure 4:**
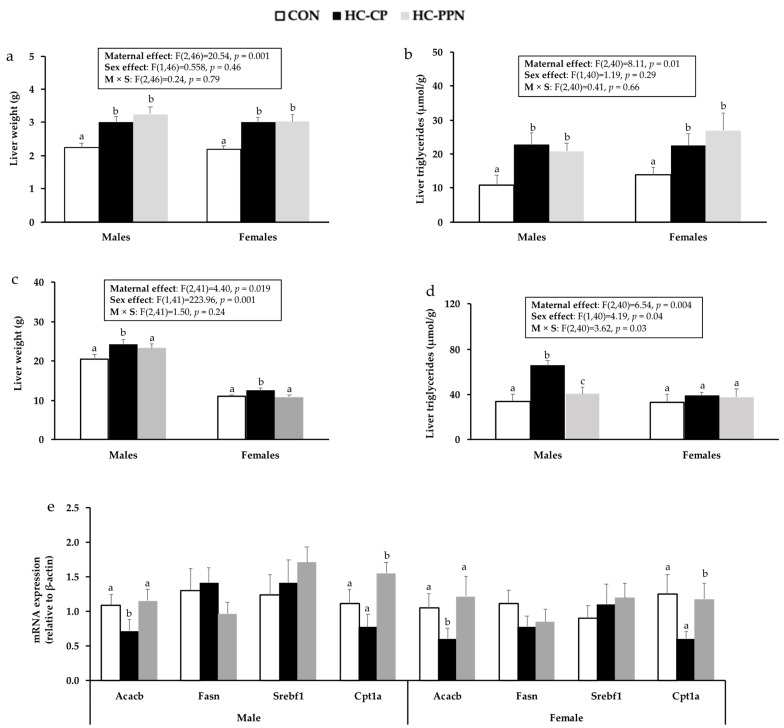
Liver outcomes in male and female offspring from CON, HC, and HC-PPN mothers including liver weights and triglyceride concentration in newly weaned (**a**,**b**) and adult (**c**,**d**) offspring; mRNA expression of lipid-regulatory targets in adult offspring (**e**). CON, low-calorie control diet; HC-CP, high-calorie obese-inducing diet with casein protein; and HC-PPN, HC high-calorie diet with supplemental yellow pea protein (25%). Maternal (M), sex (S), and interaction (M × S) were analyzed using two-way ANOVA with LSD post hoc test (*p* < 0.05). ^abc^ Treatment groups within sex that do not share a superscript are significantly different (*p* < 0.05). Data are means ± SE, and n = 7–9 mothers per group.

Liver weights in HC-CP and HC-PPN pups on PND21 were higher (*p* < 0.05) than in CON pups but did not differ from each other (Figure 4a). In adult animals, male and female pups from HC-CP dams demonstrated higher (*p* < 0.05) liver weights (vs. CON) that were normalized to CON levels by maternal YPPN supplementation (Figure 4c). Compared with CON, liver TG was increased (*p* < 0.05) to a similar extent in both newly weaned males and females from HC-CP and HC-PPN dams (Figure 4b). In adult male but not female offspring, liver TG was increased in HC-CP offspring (vs. CON) and reduced in HC-PPN offspring (vs. HC-CP) (Figure 4d). No differences (*p* < 0.05) were observed in liver cholesterol concentrations between the treatment groups.

mRNA expression of Acaca was reduced (*p* < 0.05) in HC-CP offspring compared with CON offspring (males, 0.65 fold; females, 0.4 fold) but increased (*p* < 0.05) in both male (1.6 fold) and female (1.9 fold) offspring from HC-PPN mothers (Figure 4e) compared with those from HC-CP mothers. Furthermore, HC-PPN offspring demonstrated higher Cpt1a mRNA expression compared with HC-CP offspring in adulthood (Figure 4e).

## 4. Discussion

Using a rat model of maternal obesity, we assessed if the quality of maternal dietary protein consumption, as part of a high-calorie diet throughout pre-pregnancy, gestation, and lactation, influenced the metabolic programming of obesity and lipid metabolism in offspring. Male, but not female, offspring from mothers fed the HC diet with casein protein (HC-CP) demonstrated hyperphagia, obesity, dyslipidemia, and hepatic TG accumulation as adults. However, although we observed no influence of YPPN on offspring BW in early life or adulthood, isocaloric exchange of casein for YPPN (HC-PPN) in a high-calorie obese-inducing diet improved several aspects of lipid metabolism in male offspring including serum total and LDL/VLDL cholesterol, serum TG, and hepatic TG concentration. Reduced liver TG in HC-PPN vs. HC-CP offspring was associated with increased mRNA expression of both Acacb and CPT1a that regulate both lipid synthesis and oxidation, respectively.

It is worth noting that the metabolic improvements we observed in adult male offspring from HC-PPN mothers were independent of any change in maternal obesity status throughout pre-pregnancy, gestation, and lactation. This is perhaps surprising given that previous work reported that pea protein consumption protected against BW gain in diet-induced obese rats by reducing feed intake [18]. However, the majority of previous work has been conducted in male rats, and, in general, few investigations have examined the influence of pulse consumption specifically in maternal obese models. The lack of improvement in maternal obesity with YPPN supplementation may also explain why we did not observe any protective effects on offspring BW. Furthermore, despite no change in maternal obesity, HC-PPN mothers demonstrated a notable improvement in reproductive success compared with HC-CP mothers (72.7 vs. 43.7%). Maternal nutrition has been shown to greatly influence reproduction and fertility outcomes [27]. Consumption of excess refined carbohydrates can result in metabolic dysfunction including insulin resistance that may lead to hormonal and ovulatory dysfunction [28,29]. However, we observed no difference in glycemic control outcomes (glucose, insulin, and glucose:insulin ratio) between treatment groups. Although a minor amount (~2%) of maltodextrin and sucrose was removed in the HC-PPN diet to account for the carbohydrate content of the YPPN, this negligible adjustment was not likely enough to significantly alter reproductive performance. Similarly, although consumption of both high- and low-protein diets [30,31] has been reported to have adverse effects on fertility measures, the HC-CP and HC-PPN diets were formulated to have a similar macronutrient profile with 20% of energy from either animal (casein) or plant-based sources (YPPN). Alternatively, by influencing embryo implantation and development in the early stages of pregnancy, the source and quality of dietary amino acids may influence fertility outcomes [27,32]. A previous prospective study reported a 50% reduction in the risk of ovulatory infertility with the consumption of 5% total energy as vegetable versus animal protein [33]. Thus, although the mechanism is unknown at this time, results from the current study suggest that maternal YPPN consumption may be an effective strategy to improve adverse fertility issues that are commonly observed in high-fat-fed and obese rodent models [34,35].

Both the source and amount of dietary protein have been shown to influence metabolic health in previous rodent studies [36,37,38]. However, the majority of this work has been conducted in adult (mostly male) animals. In maternal models, consumption of protein-restricted diets during pregnancy and/or lactation has been shown to induce a range of metabolic complications in offspring, including stunted growth [39], pancreatic beta-cell deficiency [40], and altered organ development [41]. Interestingly, a recent study in Wistar rats suggested that metabolic dysfunction in offspring from mothers consuming insufficient and/or low-quality protein intake during the perinatal period could be reversed via the consumption of normal protein diets during the post-weaning period in offspring [42]. Similarly, excessive maternal protein consumption has been associated with both improved metabolic outcomes (i.e., sex-specific responses in glucose tolerance and obesity [16]) and adverse health responses (i.e., increased fat mass) [43]. Alternatively, relatively few studies have examined if the source of maternal dietary protein intake during pregnancy and lactation can influence offspring health. Maternal vegetable vs. animal protein consumption throughout gestation and lactation was shown to increase BW and food intake in adult male offspring fed a postnatal vegetable-based diet [44]. These responses were associated with changes in maternal milk composition including protein and leptin. We also observed changes in maternal milk composition in mothers consuming the YPPN vs. HC mothers, including reduced total energy, fat, and protein (minor).

We observed that adult male offspring from HC-CP mothers had higher body weights and food intakes than offspring from CON mothers, confirming that maternal obesity can increase the risk of obesity in offspring, at least in males. This sex-specific response has been observed in some [45,46], but not all [47,48], previous rodent model studies investigating the transgenerational impact of maternal obesity. Chang et al. (2019) reported that male mouse offspring born to mothers fed a high-fat diet before conception had greater weight gain and subcutaneous adipose mass compared with their female counterparts when exposed to a postnatal high-fat diet challenge [49]. Similarly, a long-term study (with a 12-month postnatal period) by Nivoit et al. reported hyperphagia and increased body weights in male Wistar rat offspring from obese mothers. Female offspring demonstrated a similar early trend in body weight; however, the difference was not significant and converged at week 52 [50]. Previous human studies may also support a sex-specific detrimental impact of maternal obesity on offspring disease risk. In a previous study examining the association between maternal pre-pregnancy BMI and childhood body composition, Andres et al. (2015) reported that boys, but not girls, born to obese mothers had a higher body fat composition from ages of 2 to 6 years [51]. The underlying reasons for this detrimental sex-specific response are not entirely clear, although it may be associated with the protective effects of estrogen on obesity and cardiometabolic health [52].

Carlin et al. 2020 [53] reported that maternal consumption of pea protein during gestation and lactation reduced BWs and TG (plasma and liver) in female Wistar rat offspring compared with mothers consuming cow’s milk protein. However, their model and design differed substantially from our study as the maternal diets were not obesogenic, and offspring from the pea protein groups were exposed to a postnatal model of macronutrient dietary self-selection. Nonetheless, we also observed lower hepatic TG and reduced serum LDL/VLDL cholesterol in adult male offspring from pea-protein-fed mothers. Similarly, previous studies in adult male rodents suggest that dietary pulses protein from white lupin beans improves blood lipids and reduces liver TG concentration, possibly by inhibiting hepatic SREBP1c and FAS mRNA expression [21,54]. In our study, hepatic Acaca mRNA expression was reduced in HC-CP male offspring, possibly as a negative feedback response to higher TG, but normalized to CON levels in HC-PPN males. We also observed higher Cpt1a expression in HC-PPN versus HC-CP offspring, suggesting that the reduced hepatic TG levels in this group may be mediated by an enhanced capacity for fat oxidation. However, Cpt1a mRNA was also reduced in female HC-PPN offspring without a corresponding reduction in hepatic TG. Thus, the specific mechanism(s) underlying the sex-divergent protection against dyslipidemia in male offspring from HC-PPN dams is currently not clear but may be associated with altered milk composition. Future mechanistic understanding may be advanced by examining potential changes in the maternal microbiome (within both milk and the large intestine), as protein quality has been shown to alter microbial diversity and influence a range of health outcomes in offspring [55].

## 5. Conclusions

This study examined if the exchange of casein for YPPN in an obese-inducing maternal diet throughout pregnancy and lactation altered pregnancy outcomes and offered protection from obesity and dyslipidemia in offspring. Our findings suggest that maternal YPPN consumption may be an effective strategy to improve adverse fertility issues that are commonly observed in high-fat-fed and obese rodent models. Furthermore, we observed that in the absence of any change in maternal obesity status, maternal substitution of casein for YPPN protected adult male offspring from maternal obesity-induced dyslipidemia, with improvements in blood cholesterol, serum TG, and liver TG accumulation. We conclude that maternal dietary protein quality can influence fertility outcomes and alter offspring metabolic disease risk in later life.

## Figures and Tables

**Table 1:** Experimental diet formulation (% composition).

	Experimental Diets ^1^		
**Ingredient**	**CON**	**HC-CP**	**HC-PPN**
Casein	19.0	23.3	5.3
L-Cystine	0.3	0.3	0.4
Corn starch	52.1	8.5	9.3
Maltodextrin	14.2	11.7	10.0
Sucrose	0.4	20.6	19.3
Cellulose, BW200	4.7	5.8	3.0
Yellow pea protein isolate	-	-	25.0
Soybean oil	2.4	2.9	3.0
Lard	1.9	20.7	18.5
Mineral mix	4.7	5.8	5.8
Vitamin mix	0.1	0.1	0.1
Choline bitartrate	0.2	0.2	0.2
**Energy contribution**			
Total energy (kcal/g)	3.8	4.8	4.6
% energy from fat	10.0	45.2	45.9
% energy from protein	20.1	20.1	20.7
% energy from carbohydrate	69.8	34.7	35.7

^1^ CON, low-calorie control diet based on D12450 formulation (Research Diets); HC-CP, high-calorie obese-inducing diet with casein protein, based on D12451 (Research Diets); HC-PPN, HC diet supplemented with yellow pea protein isolate (Vitessence Pulse 1803 pea protein, Ingredion). HC-PPN diet was formulated to be matched for energy and macronutrient contribution based on proximate nutrient analyses of pea protein isolate: moisture, 9.4%; ash, 4.21%; protein, 71.48%; fat, 6.78%; total carbohydrate, 8.13%; and calories, 3.38 kcal/g.

**Table 2:** Maternal phenotype and pregnancy outcomes throughout the study.

Outcome	CON	HC-CP	HC-PPN
**Body weight (g)**			
Initial	90.7 ± 6.7 ^a^	94.3 ± 2.1 ^a^	94.61 ± 1.3 ^a^
End of pre-pregnancy	291.6 ± 8.4 ^a^	372.5 ± 15.1 ^b^	386.7 ± 12.4 ^b^
End of gestation	446.9 ± 11.3 ^a^	488.3 ± 17.4 ^b^	519.6 ± 14.7 ^b^
End of lactation	336.1 ± 11.2 ^a^	345.9 ± 9.6 ^a^	355.4 ± 13.5 ^a^
**Feed intake (kcal/d)**			
Pre-pregnancy	63.3 ± 1.7 ^a^	80.2 ± 3.7 ^b^	77.3 ± 3.1 ^b^
Gestation	85.1 ± 3.1 ^a^	98.2 ± 4.5 ^b^	104.6 ± 7.3 ^b^
Lactation	124.3 ± 4.4 ^a^	130.8 ± 3.3 ^b^	132.4 ± 4.8 ^b^
**Breeding outcomes**			
Reproductive success (%) *	90.00	43.75	72.73
Time to pregnancy (days)	2.5 ± 0.5 ^a^	2.8 ± 0.4 ^a^	3.0 ± 0.3 ^a^
Litter size at birth (# pups)	14.3 ± 0.5 ^a^	14.0 ± 0.6 ^a^	13.6 ± 0.9 ^a^
Litter weight at birth (g)	88.9 ± 3.1 ^a^	88.4 ± 5.2 ^a^	93.7 ± 7.7 ^a^
Average pup weight at birth (g)	6.2 ± 0.3 ^a^	6.3 ± 0.2 ^a^	6.82 ± 0.1 ^a^
**Metabolic parameters**			
Pre-pregnancy			
Glucose (mg/dL)	76.1 ± 6.7	72.9 ± 7.4	84.3 ± 12.4
Insulin (µIU/mL)	65.7 ± 7.5	57.86 ± 14.5	47.5 ± 9.9
Glucose:insulin	1.2 ± 0.1	1.8 ± 0.4	2.3 ± 0.4

* Calculated as mothers who gave birth without subsequent infanticide. CON, low-calorie control diet; HC-CP, high-calorie obese-inducing diet with casein protein; and HC-PPN, HC high-calorie diet with supplemental yellow pea protein (25%). ^ab^ Treatment groups that do not share a superscript are significantly different (*p* < 0.05). Data are means ± SE, and n = 7–9 per group.

**Table 3:** Serum lipid profiles (mg/dL) in male and female newly weaned and adult offspring.

	Male Offspring			Female Offspring					
Outcome	CON	HC-CP	HC-PPN	CON	HC-CP	HC-PPN	Maternal Effect	Sex Effect	M × S
**Newly weaned**									
Total-C	132.79 ± 10.38 ^a^	107.4 ± 15.15 ^ab^	83.08 ± 12.30 ^b^	130.82 ± 7.23 ^a^	124.21 ± 22.14 ^ab^	107.36 ± 9.17 ^b^	0.03	0.23	0.57
HDL-C	78.93 ± 8.78 ^a^	70.23 ± 12.34 ^a^	53.54 ± 0.90 ^b^	73.94 ± 3.13 ^a^	67.87 ± 11.25 ^a^	47.73 ± 7.91 ^b^	0.02	0.40	0.99
LDL/VLDL-C	53.86 ± 9.93	50.56 ± 8.37	53.38 ± 9.21	56.88 ± 7.14	56.34 ± 11.70	53.92 ± 1.77	0.97	0.70	0.97
**Adult**									
Total-C	210.92 ± 2.85 ^a^	356.82 ± 13.30 ^b^	280.11 ± 15.24 ^c^	322.90 ± 62.49 ^a^	286.30 ± 38.86 ^a^	271.42 ± 15.88 ^a^	0.23	0.69	0.03
HDL-C	96.27 ± 15.83	120.67 ± 16.12	120.00 ± 11.05	135.71 ± 16.01	106.63 ± 8.36	108.97 ± 13.71	0.99	0.67	0.12
LDL/VLDL-C	133.50 ± 6.72 ^a^	236.16 ± 5.73 ^b^	160.11 ± 7.47 ^c^	187.19 ± 49.44 ^a^	150.00 ± 16.67 ^a^	162.45 ± 11.04 ^a^	0.32	0.61	0.03
Triglyceride	109.71 ± 5.89 ^a^	68.67 ± 23.17 ^a^	30.80 ± 8.97 ^b^	19.85 ± 5.08	32.57 ± 12.64	27.88 ± 6.05	0.048	0.001	0.018

CON, low-calorie control diet; HC-CP, high-calorie obese-inducing diet with casein protein; and HC-PPN, HC high calorie diet with supplemental yellow pea protein (25%). Maternal (M), sex (S), and interaction (M × S) were analyzed using two-way ANOVA with LSD post hoc test (*p* < 0.05). ^abc^ Treatment groups within sex that do not share a superscript are significantly different (*p* < 0.05). Data are means ± SE, and n = 7–9 mothers per group.

## Data Availability

The data presented in this study are available on request from the corresponding author.
